# A Protein-Based Genetic Screening Uncovers Mutants Involved in Phytochrome Signaling in *Arabidopsis*

**DOI:** 10.3389/fpls.2016.01086

**Published:** 2016-07-22

**Authors:** Ling Zhu, Ruijiao Xin, Enamul Huq

**Affiliations:** Department of Molecular Biosciences and The Institute for Cellular and Molecular Biology, The University of Texas at Austin, AustinTX, USA

**Keywords:** *Arabidopsis*, genetic screening, luminescent imaging, photomorphogenesis, phytochrome interacting factor

## Abstract

Plants perceive red and far-red region of the light spectrum to regulate photomorphogenesis through a family of photoreceptors called phytochromes. Phytochromes transduce the light signals to trigger a cascade of downstream gene regulation in part via a subfamily of bHLH transcription factors called Phytochrome Interacting Factors (PIFs). As the repressors of light signaling pathways, most PIFs are phosphorylated and degraded through the ubiquitin/26S proteasome pathway in response to light. The mechanisms involved in the phosphorylation and degradation of PIFs have not been fully understood yet. Here we used an EMS mutagenesis and luminescent imaging system to identify mutants defective in the degradation of one of the PIFs, called PIF1. We identified five mutants named stable PIF (*spf*) that showed reduced degradation of PIF1 under light treatment in both luminescent imaging and immunoblot assays. The amounts of PIF1 in *spf3*, *spf4*, and *spf5* were similar to a PIF1 missense mutant (PIF1–3M) that lacks interactions between PIF1 and phyA/phyB under light. The hypocotyl lengths of *spf1* and *spf2* were slightly longer under red light compared to the LUC-PIF1 control, while only *spf1* displayed weak phenotype under far-red light conditions. Interestingly, the *spf3*, *spf4*, and *spf5* displayed high abundance of PIF1, yet the hypocotyl lengths were similar to the wild type under these conditions. Cloning and characterization of these mutants will help identify key players in the light signaling pathways including, the light-regulated kinase(s) and the E3 ligase(s) necessary for the light-induced degradation of PIFs.

## Introduction

Light is one of the most important factors for plant growth and development. As a sessile photosynthetic organism, plants are acutely sensitive to any environmental changes, such as light quality and quantity. At the seed stage, most plant seeds are induced to germinate with a small amount of light penetrating through the soil. When the young seedlings reach the surface of the soil, they are exposed to more light that changes the morphology of the seedlings with short hypocotyls, erected and expanded cotyledons, elongated roots and increased chlorophyll biosynthesis in a process called photomorphogenesis. The transition from skotomorphogenesis (etiolated seedling) to photomorphogenesis (de-etiolated seedling) under red/far-red light is mainly mediated by a class of photoreceptor, called phytochrome (phy) (Rockwell et al., 2006; Bae and Choi, 2008; Wit et al., 2016). There are five phytochromes in *Arabidopsis*, phyA-phyE (Mathews and Sharrock, 1997). The family members display distinct biological functions. For example, *phyA* mutant is insensitive to far-red light for seedling de-etiolation, while phyB–phyE mainly functions under red light conditions (Franklin and Quail, 2010). All phytochromes regulate gene expression in response to red light while phyA regulates gene expression under far-red light (Quail, 2007). In addition, phytochromes also form homo- and hetero-dimers among family members and regulate photomorphogenesis (Sharrock and Clack, 2004; Clack et al., 2009).

Phytochromes are allosteric proteins in two conformers that are present in distinct subcellular compartments. The cytosolic biologically inactive form of phytochromes (Pr) undergoes allosteric changes to the active form (Pfr) that is transported into the nucleus by red and far-red light stimuli (Fankhauser and Chen, 2008; Klose et al., 2015). The conformational changes and the trans-localization of the phytochromes can trigger a series changes in gene expression (Jiao et al., 2007; Quail, 2007; Leivar and Monte, 2014). The phytochrome-mediated gene expression regulation is mediated in part by a subfamily of basic helix-loop-helix (bHLH) transcription factors called Phytochrome Interacting Factors (PIFs) (Toledo-Ortiz et al., 2003; Castillon et al., 2007; Leivar and Quail, 2011). Phytochrome Interacting Factors act as repressors in the phytochrome-mediated light signaling to prevent photomophogenesis in darkness. The light-induced phosphorylation and degradation of PIFs are necessary for the switch from skotomorphogenesis to photomorphogenesis and are dependent on the interaction of PIFs with phytochromes, at least with phyA and/or phyB (Leivar and Quail, 2011; Leivar and Monte, 2014; Xu et al., 2015).

The phosphorylation followed by the degradation of the components associated with phytochromes via the ubiquitin-proteasome system is one of the major post-translational regulations of many factors in the light signaling pathways. The mechanisms of dark-induced degradation of the positive regulators in the light signaling pathways have been extensively studied (Henriques et al., 2009; Lau and Deng, 2012; Xu et al., 2015). The positive regulator LONG HYPOCOTYL5 (HY5) is degraded in the dark but stabilized under light to promote photomorphogenesis (Osterlund et al., 2000). The unphosphorylated form of HY5 is recognized and degraded in the dark, and the degradation is mediated by the E3 Ubiquitin ligase CONSTITUTIVE PHOTOMORPHOGENESIS1 (COP1) in association with SUPPRESSOR OF PHYA-105 (SPA1–4) AND CULLIN4 (Hardtke et al., 2000; Saijo et al., 2003; Zhu et al., 2008; Xu et al., 2015). Similarly, many other positive regulars are degraded in the dark by CUL4-COP1-SPA complexes (Lau and Deng, 2012; Xu et al., 2015). Unlike the posttranslational regulations of the positive regulators, the repressors in the light signaling pathways, such as PIFs are degraded under light and stabilized under darkness. All the PIFs except PIF7 are phosphorylated and degraded in response to light (Leivar and Quail, 2011). Casein kinase II (CK2) and BRASSINOSTEROID INSENSITIVE2 (BIN2) have been reported to be involved in the phosphorylation of PIF1 and PIF4, respectively (Bu et al., 2011a,b; Bernardo-García et al., 2014). However, both CK2 and BIN2 did not phosphorylate PIFs in a light-inducible manner. Very recently, phytochrome has been shown to function as a light-regulated kinase for PIF3, consistent with the previous reports of phytochrome kinase activity (Yeh and Lagarias, 1998; Shin et al., 2016). A protein called HEMERA has been shown to be necessary for the nuclear speckle formation and degradation of PIF1 and PIF3 under continuous light conditions. HEMERA is predicted to be structurally similar to RAD23 which functions as a polyubiquitylated protein shuffler for degradation (Chen et al., 2010; Qiu et al., 2015). In addition, two studies reported Light Response Bric-a-Brack/Tramtrack/Broad (BTB) proteins (LRBs) and the CUL4-COP1-SPA complex functioning as E3 Ubiquitin ligases mediating PIF3 and PIF1 degradation upon light exposure, respectively (Ni et al., 2014; Xu et al., 2015; Zhu et al., 2015). However, in both cases, PIFs are still degraded under prolonged light treatment, suggesting other factors are necessary for PIF degradation in response to light.

Another type of post-translational regulation for the bHLH proteins such as PIFs is heterodimerization with non-DNA binding HLH proteins to inhibit the DNA binding and transcriptional activation activity of PIFs. There are 162 bHLH proteins in *Arabidopsis* of which at least 27 are predicted to be non-DNA binding proteins (Toledo-Ortiz et al., 2003). The HLH proteins have been shown to prevent the DNA binding activity of the bHLH transcription factors in plants (Hornitschek et al., 2009; Zhang et al., 2009; Shi et al., 2013; Zhu et al., 2016). In light signaling pathways, the antagonistic function of the HLH–bHLH also mediates the seed germination and shade avoidance responses (Hornitschek et al., 2009; Shi et al., 2013). We have recently identified a family of HLH proteins called HECATEs (HEC1, HEC2, and HEC3) that lack the DNA binding basic domain (Zhu et al., 2016). HEC2 can block the DNA binding activity of PIF1, which results in decreased transcriptional activation of PIF1 target genes. Strikingly, HEC2 also prevents the light-induced degradation of PIF1. It is possible that the heterodimers may not be recognized by either the kinase and/or the E3 ligase for the light-induced phosphorylation and degradation via the proteasome machinery. As a result, HLH proteins may regulate not only the DNA binding activity of the bHLH factors but also their protein abundance.

Many mutants defective in the light signaling pathways have been identified by genetic screening based on the visible morphological phenotypes (Koornneef et al., 1980; Chory et al., 1989; Deng et al., 1992; Huq and Quail, 2005). However, a mutation affecting both the positive and negative regulators may not display visible morphological phenotypes due to the balance between the positive and negative regulatory functions. Neither the LRBs nor the CUL4-COP1-SPA complex responsible for the PIF degradation was identified through the traditional genetic screening methods. Because both PIF1 and PIF3 are still degraded in the *cul4-cop1-spa* and *lrb* mutants, respectively; it is still possible that there are other kinases and E3 ligases functioning on different PIFs at various stages of the life cycle. Therefore, new genetic screens based on the protein level as opposed to the visible morphological phenotypes are necessary, which can precisely monitor PIF abundance.

Genetic screening by the bioluminescent imaging method has been used to identify mutants in the circadian clock regulation and abiotic stress signaling pathways (Millar et al., 1992; Chinnusamy et al., 2002; Lee et al., 2002; Onai et al., 2004). In addition, a transcriptional Luciferase (LUC) fusion strategy has been used to isolate mutants defective in shade avoidance responses (Wang et al., 2011). Here we used a translational fusion of *LUC (firefly)-PIF1* driven by 35S promoter to isolate mutants defective in light signaling pathways. We mutagenized *35S:LUC-PIF1* transgenic plants by ethyl methanesulfonate (EMS) and monitored the LUC-PIF1 fusion protein level through luminescent imaging. The main focus of this study is to identify mutants that have altered level of PIF1 protein. In consequence, the factors defective in those mutants are candidates specifically responsible for either the phosphorylation and/or degradation of PIF1. From this genetic screen, we have isolated one mutant that showed stable PIF1 in luminescent imaging. This mutant contains an early stop codon in the *PHYB* gene and exhibited elongated hypocotyl phenotypes under continuous red and white light conditions. Five other mutant lines showed stable PIF1 both in the luminescent imaging and immunoblot assays. These mutants displayed very weak, if any, morphological phenotypes under continuous red and far-red light conditions, yet they displayed high abundance of PIF1. Thus, the identification and characterization of these genes will provide new information on the mechanisms of light-induced degradation of PIFs and phytochrome signaling networks.

## Materials and Methods

### EMS Mutagenesis

Approximately, 40,000 seeds of LUC-PIF1 transgenic line have been mutagenized with Ethyl Methane Sulfonate (EMS). Briefly, the seeds were washed in 0.1% Tween 20 for 15 min and then slowly rotated in 0.3% EMS in a 50 ml falcon tube for 14 h in the dark followed by washing with 50 ml distilled water for three times. The seeds were finally washed with another 50 ml distilled water with 2 h rotation, and then re-suspended in 10 ml 0.1% agar. The seeds were equally distributed onto 162 2 × 2 pots by using Eppendorf repeat pipette. Each pot contained 1 ml 0.1% agar with seeds. The seeds were then stratified at 4°C for 4 days, and grown in the greenhouse to maturity. Seeds were harvested into ∼162 M2 families for screening.

### Luciferase Imaging

Approximately 100 seeds from each bulk were grown on a 100 mm × 100 mm × 15 mm square Petri dish vertically for 3 days in the dark, exposed to white light for 15 min, and then Luciferin solution (1 mM Luciferin + 0.01% Triton X-100) were sprayed on the seedlings. The plates remained in the dark for another 5 min before being imaged by the NightOWL camera (Berthold Technologies GmbH & Co. KG, Germany). The luminescent signal picture and the regular picture taken under white light have been combined together by the software Photoshop. The pseudo colors, red for the luminescent signal and green for the brightfield picture, were added by the same software for visualization.

### Plant Growth Conditions and Phenotypic Assays

Plants were grown under constant white light at 22°C for the regular growth and maturity. Seeds were surface sterilized and plated on Murashige–Skoog (MS) growth medium (GM) containing 0.9% agar without sucrose (GM-Suc) as described (Shen et al., 2005). Seeds were stratified at 4°C in the dark for 4 days, and exposed to 3 h white light at room temperature to induce germination before placing them in the dark for additional 21 h. The plates were then either placed in the dark or under specific wavelengths of light for additional 3 days. Two seedlings from each condition were picked up and lined up on a 0.9% agar plate for taking photographs.

### Protein Extraction and Immunoblot

Protein extraction and immunoblotting were performed essentially as described (Shen et al., 2005, 2008). Four-day-old dark-grown seedlings were either kept in the dark or exposed to pulses of red light followed by incubation in the dark for various times as indicated on each figure before protein extraction. To detect LUC-PIF1 proteins in transgenic plants, ∼0.2g tissue from 4-day-old dark grown seedlings were ground in 0.8 ml of boiling denaturing buffer (100 mM MOPS, pH 7.6, 5% SDS, 10% Glycerol, 4 mM EDTA, 40 mM β-mercaptoethanol, 2 mM PMSF, 1X protease inhibitor for plant cell and tissue extracts [Cat. No. P9599, Sigma–Aldrich]) and boiled for 5 min. Around 30 μg of total proteins for each sample were separated on 6% SDS-PAGE gels, blotted onto PVDF membrane and probed with anti-LUC (Promega, Madison, WI) and anti-RPT5 (Enzo Life Sciences, Farmingdale, NY, USA) antibodies.

### Statistical Analyses

Two-way ANOVA was performed for both quantification of immunoblots and hypocotyl length measurements. After two-way ANOVA analysis rejected the homoscedasticity hypothesis in both cases, Tukey’s range test was conducted as *post hoc* analysis to determine which group(s) are different from other groups under one specific condition with only one variable. Wild type and all mutants were assorted into different groups based on the *P* values under 5 or 10 min after Rp conditions for immunoblots analysis; or dark, Rc and FRc conditions for hypocotyl length measurements, specifically. Small letters indicating statistically significant difference (*p* ≤ 0.05) were assigned for each figure based on these analyses.

## Results

### The Strategy of EMS Mutagenesis and Mapping by Luciferase Imaging

**FIGURE 1:**
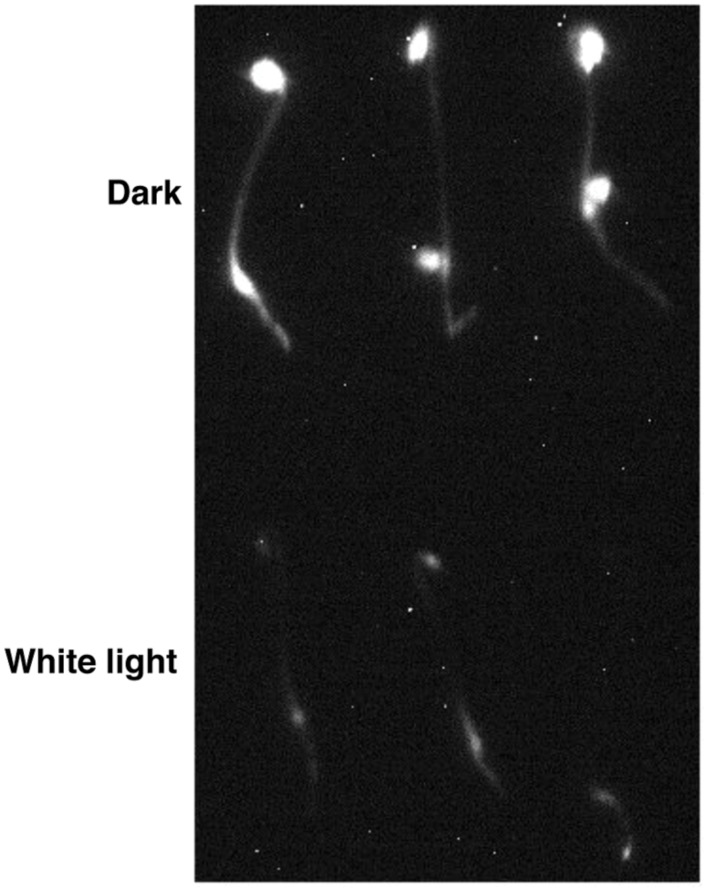
***LUC-PIF1* seedlings showed reduced luminescent signal upon white light exposure compared with dark grown seedlings.** Three-day-old dark grown *LUC-PIF1* transgenic seedlings were either kept in the dark or exposed to white light for 15 min. One millimeter Luciferin solution was sprayed on the seedlings. The plates remained in the dark for another 5 min before being imaged by the NightOWL camera.

Previously, we have described the characterization of the *35S:LUC-PIF1* transgenic lines and demonstrated the protein dynamics under red and far-red light conditions (Shen et al., 2005). We have also exposed these seedlings under white light and observed strong reduction in LUC signal within 10–15 min of white light treatment (**Figure 1**). This strong reduction of LUC-PIF1 is the basis of a genetic screening to identify mutants defective in this process.

**FIGURE 2:**
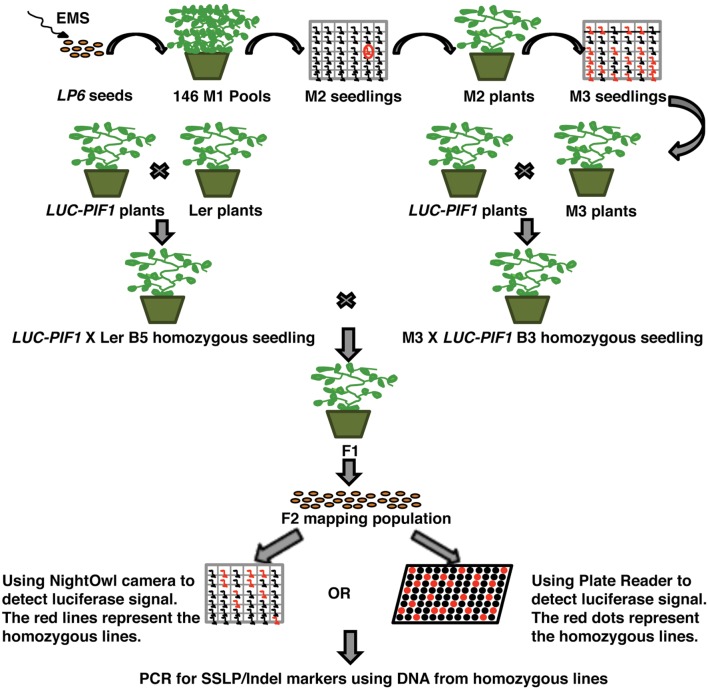
**Schematic diagram of the EMS mutant screening by luciferase imaging.** Briefly, ∼40,000 *LUC-PIF1* transgenic seeds were mutagenized with EMS, equally distributed into 162 pots for growth and harvested in 162 bulks. Hundred seeds from each bulk were tested by luciferase imaging as described (Chinnusamy et al., 2002). The seedlings showing stable luminescent signal after 15 min of white light exposure were selected and grown to maturity. Seedlings were back crossed with the parental line *LUC-PIF1* three times. The homozygous lines after three times backcrosses were outcrossed with the homozygous *LUC-PIF1* seedlings that have been introgressed into Ler by five times crossing. The F2 generation represents the mapping population used for rough mapping using SSLP/CAPS/Indel markers.

To identify mutants, ∼40,000 transgenic *35S:LUC-PIF1* homozygous seeds have been mutagenized. The mutated seeds were equally distributed into 162 2 × 2 pots, which represented 162 M2 bulks. Each bulk progeny has been harvested together. One hundred seeds from each bulk were plated to check the luminescent signal under NightOwl camera (Berthold Technologies GmbH & Co. KG, Germany). Approximately, 16,200 M2 seedlings (100 seeds/M2 family) have been investigated for the stability of LUC-PIF1 through the luminescent imaging method. Initially, we identified ∼1000 lines displaying increased LUC-PIF1 signal. Upon secondary screening in the next generation, we narrowed down to 350 lines displaying stable LUC-PIF1 activity. These 350 lines belong to 36 M2 families. Thus, we selected 10 best lines at the M4 stage. All these mutants were back-crossed with the parental *35S:LUC-PIF1* line three times and the homozygous mutants were isolated (**Figure 2**).

### Five Best Mutant Lines Showed Slow Degradation of LUC-PIF1

**FIGURE 3:**
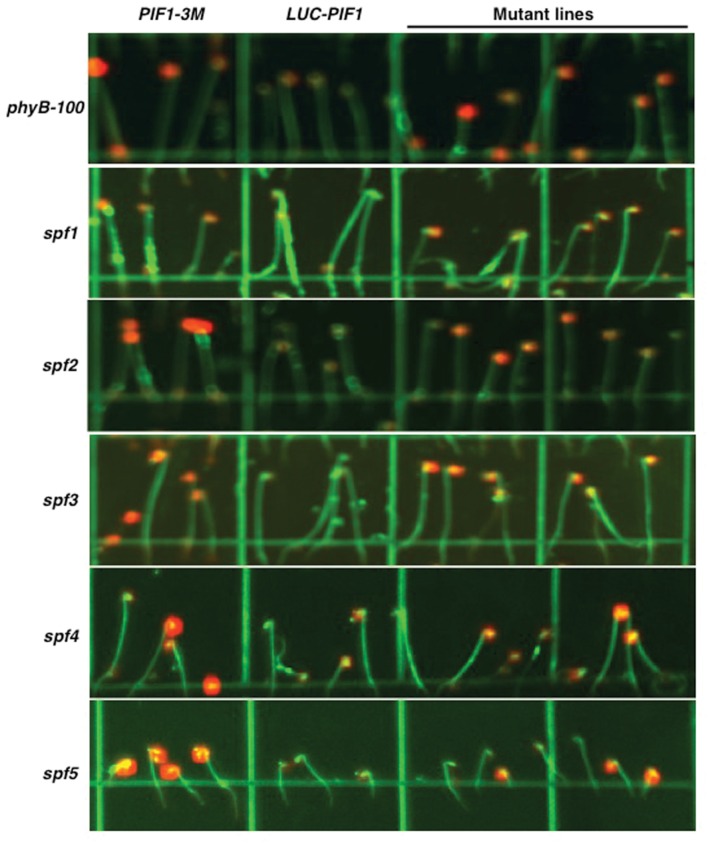
**Luciferase imaging to identify five best lines showing stable LUC-PIF1 fusion protein level.** Representative images for various mutant lines are shown along with controls. Seeds were plated on 100 mm × 100 mm × 15 mm square plates with MS media and imbibed at 4°C in darkness for 4 days. The plates were exposed to 3 h of continuous white light to stimulate germination and then kept in the dark at 21°C for 3 days. The luciferase images were taken after 15 min of white light exposure followed by 5 min treatment with 1 mM Luciferin plus 0.01% Triton X-100. Each plate contains *PIF1-3M* as a positive control and *LUC-PIF1* as a negative control. *spf5* is heterozygous in this image.

Among the 10 lines at M4 stage, six lines inherited the mutation (s) after backcrossing. All of them showed stronger luminescent signal after 15 min of continuous white light treatment compared with *35S:LUC-PIF* parental line (**Figure 3**). LUC-PIF1 has been reported to have a half-life of 15 min after 3000 μmol⋅m^-2^ red light treatment (Shen et al., 2005). Based on the luminescent signal captured by the Night Owl camera, LUC-PIF1 fusion protein is degraded fully after 15 min of continuous white light in *35S:LUC-PIF1* transgenic seedlings. LUC-PIF1 is more stable in all six EMS mutant lines than the *35S:LUC-PIF1* transgenic line. Three of them, *spf3*, *spf4*, and *spf5*, showed the same signal level as PIF1-3 M transgenic line which has been shown to have very stable PIF1 protein under light due to the lack of key amino acid residues necessary for interactions with phyA and phyB (Shen et al., 2008).

To examine if any of these mutants have intragenic mutation, we amplified the LUC-PIF1 open reading frame by PCR and sequenced them. Results showed that all six mutants did not contain any intragenetic mutation in the LUC-PIF1 sequence. One line showed similar phenotype to phyB mutant at the adult stage in greenhouse growth conditions. Sequencing of the *PHYB* gene showed that the mutant contains two mutations in the *PHYB* gene: CAG(Q489) to TAG(stop codon) and CAG(Q564) → CAT(H). The first mutation introduced an early stop codon in the *PHYB* gene and results in a truncated version of phyB. We named this mutant allele as *phyB-100*.

### *spf* Mutants Are Involved in the Degradation of PIF1, not the Light Induced Phosphorylation of PIF1

**FIGURE 4:**
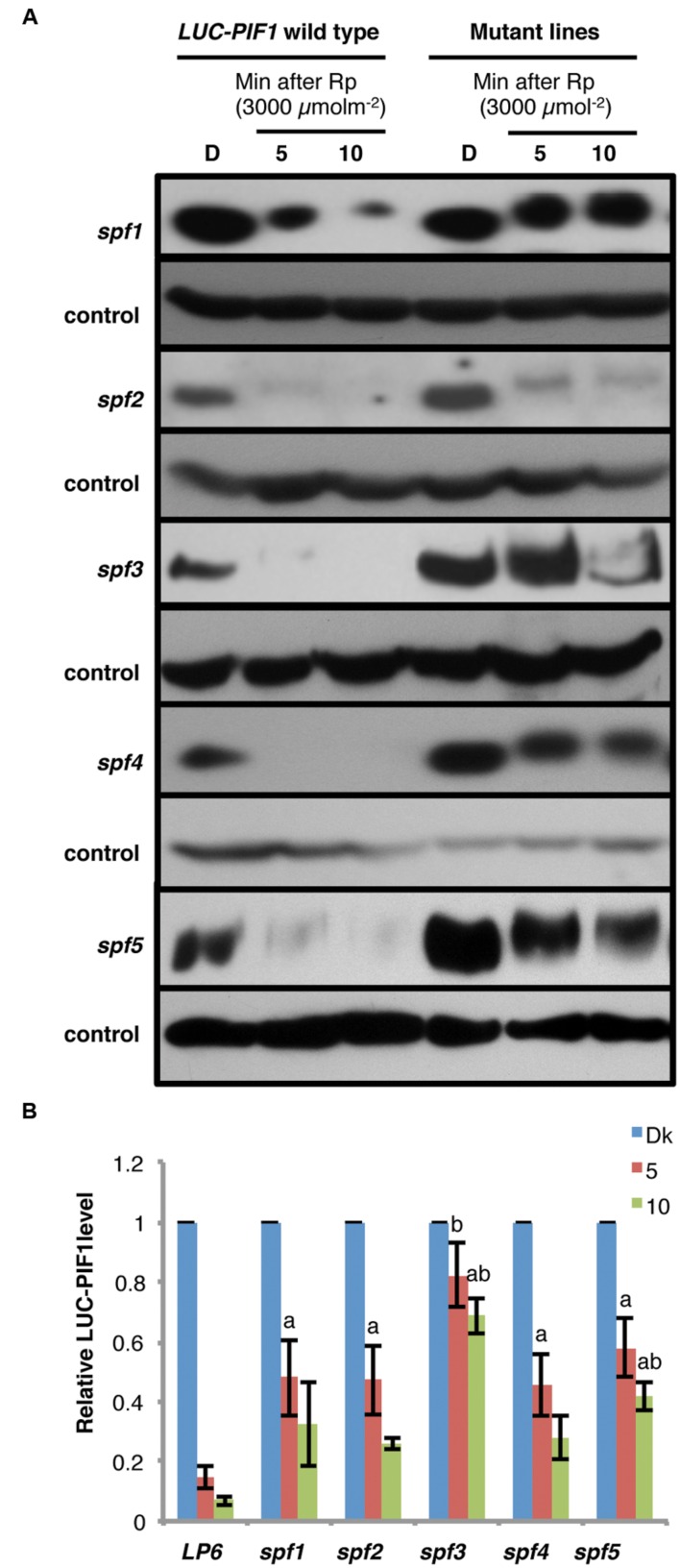
**Immunoblots showing slower degradation kinetics of LUC-PIF fusion proteins in the *spf* mutant lines compared to the LUC-PIF1 control.** **(A)** Four-day-old dark-grown seedlings of the mutants and the *LUC-PIF1* were either kept in darkness or given 3000 μmolm^-2^ red light pulse followed by different durations of dark incubation as indicated above. The total protein of the mutants and the *LUC-PIF1* were extracted from each time point and loaded into 6% SDS-PAGE gel for immunoblot analyses. The LUC-PIF1 fusion protein was detected by the primary antibody against luciferase and visualized using chemiluminescence method. **(B)** Quantification of LUC-PIF1 (LP6) level in the conditions indicated in **(A)**. Three biological repeats were performed. The band intensities were measured using ImageJ. The LUC-PIF1 protein level in each sample has been normalized using the control bands as indicated. Error bars = SEM. a, indicates statistically significant difference from *LUC-PIF1* with 5 min dark incubation after Rp; b, indicates statistically significant difference from the rest of samples with 5 min of dark incubation after Rp; ab, indicates statistically significant difference from rest of the samples with 10 min dark incubation after Rp (*p* ≤ 0.05).

There are two distinct steps in PIF degradation under light. First, PIFs are phosphorylated in response to light, and then ubiquitinated by E3 Ubiquitin ligase(s) before being degraded through the 26S proteasome pathway. To distinguish if these mutants are defective in the light-induced phosphorylation and/or ubiquitination, the fusion protein level and the mobility shift of LUC-PIF have been verified by immunoblot analyses. All five mutants showed slower degradation of LUC-PIF1 than the LUC-PIF1 control after 3000 μmolm^-2^ of red light pulse (**Figures 4A,B**). The dark level of LUC-PIF1 was higher in all five mutants compared to control (**Figure 4A**). *spf2* displayed only slight inhibition of degradation of LUC-PIF1. *spf1, spf4*, and *spf5* displayed similar level of stabilized LUC-PIF1 protein under these conditions. The quantitative data suggest that *spf3* is the most promising mutant line containing the mutation(s) in the gene involved in the rapid degradation of PIF1 upon light exposure (**Figure 4B**).

The immunoblot data also showed that even though the degradation of LUC-PIF1 was reduced, the LUC-PIF1 in these mutants are still phosphorylated after pulses of red light characterized by the band shift in response to light exposure (**Figure 4A**). Among all five mutants, only *spf3* displayed a minor defect in band shift. However, further analysis is necessary to draw conclusion if *spf3* is defective in a potential kinase as genetic redundancy might play a role in this process. Overall, these data suggest that all these mutants identified so far might be responsible for the light-induced degradation of PIF1.

### No Severe Growth Defects Were Observed in the *spf* Mutants

**FIGURE 5:**
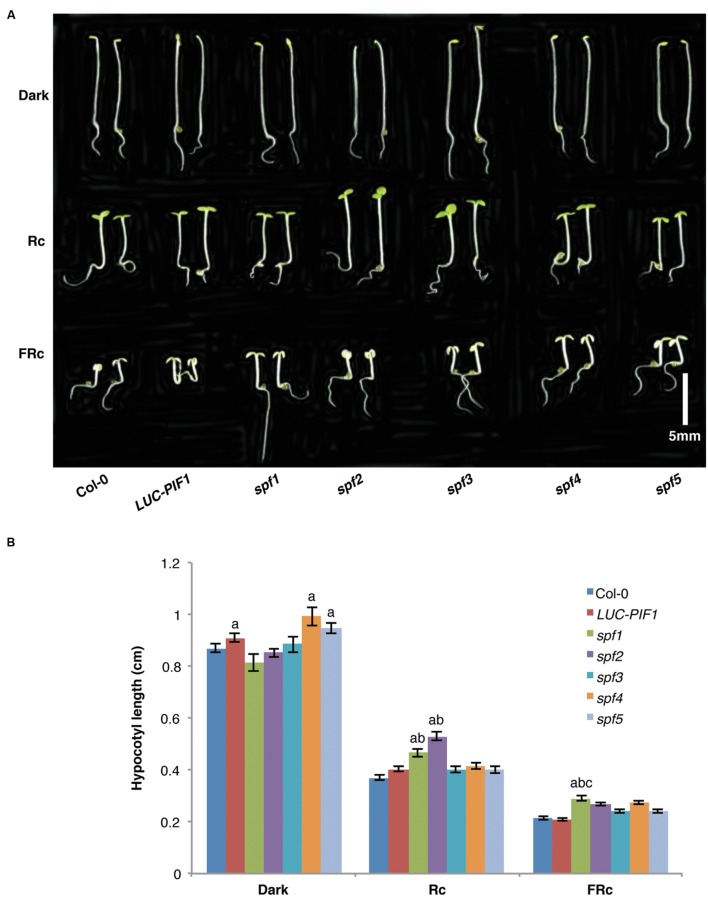
**The seedling de-etiolation phenotypes of the *spf* mutants and the control lines.** **(A)** Seeds from each line were plated on MS media and stratified at 4°C in darkness for 4 days. Three hours of white light treatment was used to induce seed germination. The plates were then wrapped with aluminum foil and kept at 21°C for 21 h. Then they were grown under either 6 μmolm^-2^s^-1^ red light, or 1 μmolm^-2^s^-1^ far-red light, or kept in darkness for additional 3 days. Bar = 0.5 cm. **(B)** Bar graph shows the hypocotyl lengths of wild-type Col-0, *LUC-PIF1*, *spf1*, *spf2 spf3*, *spf4*, and *spf5* mutants grown under conditions described above. At least 40 seedlings were measured using ImageJ for each genotype under each condition. Error bars = SE. a, indicates statistically significant difference from rest of the samples in darkness; ab, indicates statistically significant difference from rest of the samples under Rc condition; and abc, indicates statistically significant difference from rest of the samples under FRc condition (*p* ≤ 0.05).

The stabilized PIF1 under light conditions might reduce/prevent photomorphogenesis. In consequence, the mutants might display de-etiolation phenotypes at the seedling stage or adult stage. We have examined hypocotyl lengths for these mutants grown under continuous red and far-red light and in darkness. All five mutants did not display any severe growth defects. They are normal at the adult stage when grown in the growth room under continuous white light. At the seedling stage, only *spf1* and *spf2* displayed longer hypocotyls under continuous red light conditions compared with LUC-PIF1 seedlings (**Figures 5A,B**). Under continuous far-red light, *spf1* displayed slightly longer hypocotyl compared to LUC-PIF1 control (**Figure 5B**). The increased abundance of PIF1 and possibly other PIFs may contribute to this phenotype. It is surprising that *spf3*, *spf4*, and *spf5* did not display any hypocotyl phenotype under these conditions. However, further phenotypic characterization is necessary to examine if they are defective in any other light regulated processes.

## Discussion

The PIF proteins are one of the best characterized families of negative regulators in phy-mediated light signaling pathways (Leivar and Quail, 2011; Leivar and Monte, 2014; Xu et al., 2015). The identification of PIFs provided a simple linear biochemical pathway for light signal transduction, where phytochromes directly interact with and transduce the photo signals to transcription factors in response to light and regulate downstream target gene expression (Leivar and Quail, 2011). All PIFs except PIF7 are rapidly phosphorylated and ubiquitinated *in vivo* prior to their degradation in response to light (Shen et al., 2005, 2007; Al-Sady et al., 2006). Structure-function relationship studies showed that phytochrome interaction with PIFs is necessary for the light-induced phosphorylation and subsequent degradation of PIFs through the 26S proteasome pathway (Al-Sady et al., 2006; Shen et al., 2008). Many studies have been conducted to understand phytochromes-PIFs interaction and PIFs regulation on downstream target genes, but little is known about the factors necessary for the degradation of PIFs until recently, which is the key step in phytochrome-mediated light signaling pathway.

Genetic and biochemical approaches have identified three classes of mutants that showed stable PIFs under light. The first class is the photoreceptors themselves, phytochromes. Different phytochromes induce the degradation of PIFs with differential kinetics under red and/or far-red light conditions (Al-Sady et al., 2006; Shen et al., 2008). The kinetics of degradation of different PIFs largely reflect their affinities toward phyA and/or phyB (Al-Sady et al., 2006; Shen et al., 2008). This is not surprising as phytochromes are the photoreceptors that perceive light signals to induce the degradation of PIFs. Very recently, it was shown that phytochromes directly phosphorylate PIFs and regulate their degradation (Shin et al., 2016). The second class only contains one member, called *hemera.* HEMERA is necessary for the light-induced degradation of PIF1 and PIF3 under de-etiolated conditions (Chen et al., 2010; Qiu et al., 2015). The homolog of HEMERA in yeast, RAD23, is a multiubiquitin-binding protein, functions in carrying polyubiquitylated proteins to the 26S proteasome for degradation. The third class involves E3 Ubiquitin ligases (e.g., CUL3-LRBs and CUL4-COP1-SPA complex) functioning in the light-induced ubiquitination of PIFs (Ni et al., 2014; Xu et al., 2015; Zhu et al., 2015). In addition, two kinases (CK2 and BIN2) have been shown to phosphorylate PIFs and this phosphorylation enhances PIF degradation in response to light (Bu et al., 2011a; Bernardo-García et al., 2014). However, PIFs are still degraded in these mutant backgrounds, suggesting other factors are necessary for their degradation.

To identify factors regulating PIF abundance, we have taken an unbiased non-invasive LUC imaging-based genetic screen to identify mutants affecting PIF1 stability in response to light. This protein-based approach has several advantages over conventional hypocotyl length-based genetic screens. Firstly, the non-invasive feature of this method provides the possibility to monitor the luminescent signal in every step of the mapping procedure and allows seedlings to grow to the next generation after imaging. Since the dark-grown seedlings are much weaker than light-grown seedlings, non-invasive imaging is very important to obtain the progeny of the possible mutants. Secondly, because of the rapid degradation of PIF1, the LUC reporter system can detect very low level of PIF1 protein in a real time manner that may not be detected using immunoblots. Thirdly, the luminescent signal is also quantitative and the quantitative data can be used in the mapping process to distinguish homozygous and heterozygous mutants. The luminescent imaging method can be universally used for studying posttranslational regulation of any proteins. Fourth and most importantly, this method allows the identification of mutants based on protein level as opposed to the morphological phenotypes. Because PIF1 functions in the early steps during dark to light transition, the mutants containing partially stable PIF1 may not show any hypocotyl phenotype. Those mutants might be screened out in the traditional hypocotyl-based genetic screens (Koornneef et al., 1980; Chory et al., 1989; Deng et al., 1992). Also, because PIF1 prevents seed germination, mutants resulting in fully stable PIF1 under light may not germinate. Our screen has identified five extragenic mutants that are involved in the degradation of PIF1. However, only two, *spf1* and *spf2*, showed slightly longer hypocotyls under red light compared to the LUC-PIF1 seedlings, while *spf1* displayed a weak phenotype under far-red light conditions. The *spf3* mutant displayed the most stable LUC-PIF1 protein level in both luminescent imaging and immunoblot assays. However, *spf3, spf4*, and *spf5* did not display any visible phenotypes either at the seedling stage or at the adult stage. These mutants would have been eliminated based on the conventional hypocotyl-based genetic screens. Identification of these mutants highlights the power of the luminescent imaging screening method compared to the traditional genetic screening methods employed in light signaling field.

Combining phenotypic analyses with the powerful LUC imaging technique allowed us to categorize the mutants that showed visible phenotypes under monochromatic light conditions as well as LUC-PIF1 stability vs. only LUC-PIF1 stability without any discernible phenotypes. The mutants that did not display any hypocotyl phenotypes (e.g., *spf3*, *spf4*, and *spf5*) represent a novel class of mutants that displayed molecular phenotypes without any visible phenotypes. It is possible that these mutants are defective in the degradation of both positive and negatively acting factors involved in light signaling pathways, resulting in the balancing out of the effects. Alternatively, they might also have defects in other pathways that oppose light signaling pathways. In summary, this study highlights the use of the LUC-based genetic screening method to identify mutants in light signaling pathways. It shows the isolation and preliminary characterization of five mutants involved in PIF1 degradation in response to light. However, much remains to be learned about these mutants including complementation analyses, map locations and the genes defective in these mutants. Further phenotypic characterization of these mutants including examination of other PIF levels is necessary to understand which processes are defective in these mutants. In addition, examination of native PIF levels in these mutants are necessary to rule out any artifacts due to the use of 35S:LUC-PIF1 transgenic lines. Finally, cloning of these genes will contribute to better understanding of the networks of phytochrome-mediated light signaling pathways.

## Author Contributions

LZ, RX, and EH designed experiments. LZ and RX carried out experiments. LZ, RX, and EH analyzed data. LZ and EH wrote the manuscript; RX commented on the manuscript.

## Conflict of Interest Statement

The authors declare that the research was conducted in the absence of any commercial or financial relationships that could be construed as a potential conflict of interest.
